# 1-(3-Bromopropoxy)-4-chlorobenzene

**DOI:** 10.1107/S1600536808037896

**Published:** 2008-11-22

**Authors:** Wen-ge Yang, Jin-feng Yao, Xiao-lei Zhao, Yong-hong Hu

**Affiliations:** a College of Life Science and Pharmaceutical Engineering, Nanjing University of Technology, Xinmofan Road No. 5 Nanjing, Nanjing 210009, People’s Republic of China

## Abstract

In the mol­ecule of the title compound, C_8_H_8_BrClO, the Cl atom lies slightly out of the aromatic ring plane [displacement = 0.072 (3) Å]. In the crystal structure, a π–π contact between the phenyl rings [centroid–centroid distance = 3.699 (3) Å] may stabilize the structure. There also exists a C—H⋯π contact between the methyl­ene group and the chloro­phenyl ring.

## Related literature

For general background, see: Zirngibl *et al.* (1988 ▶). For related structures, see: Menini & Gusevskaya (2006 ▶); Baggaley & Watts (1982 ▶). For bond-length data, see: Allen *et al.* (1987 ▶).
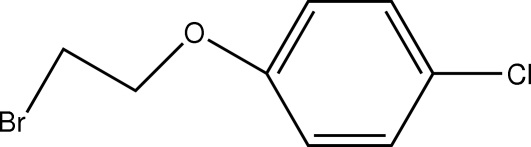


## Experimental

### 

#### Crystal data

C_8_H_8_BrClO
*M* *_r_* = 235.50
Monoclinic, 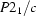

*a* = 9.0680 (18) Å
*b* = 9.781 (2) Å
*c* = 10.238 (2) Å
β = 98.01 (3)°
*V* = 899.2 (3) Å^3^
*Z* = 4
Mo *K*α radiation
μ = 4.81 mm^−1^
*T* = 294 (2) K
0.30 × 0.20 × 0.20 mm

#### Data collection

Enraf-Nonius CAD-4 diffractometer
Absorption correction: ψ scan (North *et al.*, 1968 ▶) *T*_min_ = 0.327, *T*_max_ = 0.382
1726 measured reflections
1620 independent reflections
769 reflections with *I* > 2σ(*I*)
*R*_int_ = 0.060
3 standard reflections frequency: 120 min intensity decay: 1%

#### Refinement

*R*[*F* ^2^ > 2σ(*F* ^2^)] = 0.078
*wR*(*F* ^2^) = 0.166
*S* = 1.00
1620 reflections
100 parameters
H-atom parameters constrained
Δρ_max_ = 0.48 e Å^−3^
Δρ_min_ = −0.51 e Å^−3^

### 

Data collection: *CAD-4 Software* (Enraf–Nonius, 1989 ▶); cell refinement: *CAD-4 Software*; data reduction: *XCAD4* (Harms & Wocadlo, 1995 ▶); program(s) used to solve structure: *SHELXS97* (Sheldrick, 2008 ▶); program(s) used to refine structure: *SHELXL97* (Sheldrick, 2008 ▶); molecular graphics: *PLATON* (Spek, 2003 ▶); software used to prepare material for publication: *SHELXTL* (Sheldrick, 2008 ▶).

## Supplementary Material

Crystal structure: contains datablocks Y, I. DOI: 10.1107/S1600536808037896/hk2572sup1.cif

Structure factors: contains datablocks I. DOI: 10.1107/S1600536808037896/hk2572Isup2.hkl

Additional supplementary materials:  crystallographic information; 3D view; checkCIF report

## Figures and Tables

**Table 1:** Hydrogen-bond geometry (Å, °)

*D*—H⋯*A*	*D*—H	H⋯*A*	*D*⋯*A*	*D*—H⋯*A*
C2—H2*A*⋯*Cg*1^i^	0.97	2.88	3.665 (3)	138

Symmetry code: (i) 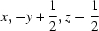. *Cg*1 is the centroid of the C3–C8 ring.

**Fig. 1.**
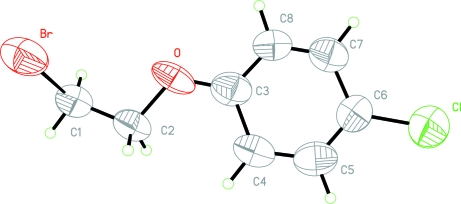
The molecular structure of the title molecule, with the atom-numbering scheme. Displacement ellipsoids are drawn at the 30% probability level.

## supplementary crystallographic information

### Comment

Omoconazole has a high antifungal activity and a broad spectrum (Zirngibl *et al.*,  1988). As part of our ongoing studies in this area, we report herein the crystal structure of the title compound.

In the molecule of the title compound (Fig. 1) the bond lengths (Allen *et al.*,  1987) and angles are within normal ranges. Ring A (C3-C8) is, of course, planar, and the Cl atom lies slightly out of the ring plane [0.072 (3) Å]. The (O1/C1/C2) and (Br/C1/C2) moieties are oriented with respect to ring A at dihedral angles of 11.57 (3)° and 74.97 (3)°, respectively.

In the crystal structure, the π-π contact between the phenyl rings, Cg1—Cg1^i^ [symmetry code: (i) -x, 1 - y, -z, where Cg1 is centroid of the ring A (C3-C8)] may stabilize the structure, with centroid-centroid distance of 3.699 (3) Å. There also exists a C—H···π contact (Table 1) between the methylene group and the chlorophenyl ring.

### Experimental

Phenol (47.0 g, 0.5 mol), CuCl_2_ (147.4 g, 1.1 mol) and hydrochloric acid (350 ml, 8.5 mol/L) were mixed in a three-necked flask equipped with a reflux condenser and a magnetic stirrer. The solution was stirred at 383 K for 10 h, and then cooled to room temperature. Subsequently the reaction mixture was extracted with toluene for three times, and then the extracts were dried and the solvents were completely stripped by evaporation. After isolated by column chromatography (silica), p-chlorophenol was obtained (yield; 44.8 g, 75%) (Menini & Gusevskaya, 2006). p-Chlorophenol (26.0 g, 0.2 mol) was dissolved with stirring in water (30 ml) containing sodium hydroxide (9.0 g, 0.23 mol) and added dropwise to excess refluxing ethylene dibromide (74.8 g, 0.4 mol). The reaction mixture was heated under reflux for 6 h, cooled and extracted into ether (3 *x* 150 ml). The combined organic extracts were washed with water, dried over Na_2_S0_4_, filtered and evaporated to dryness to yield an oil. Fractionation under reduced pressure yielded p-chlorophenoxyethyl bromide as a colorless oil, then cooled to give the title compound as colorless solid (yield; 27.6 g, 57%) (Baggaley & Watts, 1982). Crystals suitable for X-ray analysis were obtained by slow evaporation of an petroleum ether solution.

### Refinement

H atoms were positioned geometrically, with C-H = 0.93 and 0.97 Å for aromatic and methylene H, respectively, and constrained to ride on their parent atoms with U_iso_(H) = 1.2U_eq_(C).

### Crystal data

**Table:**

C_8_H_8_BrClO	*F*_000_ = 464
*M**_r_* = 235.50	*D*_x_ = 1.740 Mg m^−^^3^
Monoclinic, *P*2_1_/*c*	Mo *K*α radiation λ = 0.71073 Å
Hall symbol: -P 2ybc	Cell parameters from 25 reflections
*a* = 9.0680 (18) Å	θ = 10–14º
*b* = 9.781 (2) Å	µ = 4.81 mm^−^^1^
*c* = 10.238 (2) Å	*T* = 294 (2) K
β = 98.01 (3)º	Block, colorless
*V* = 899.2 (3) Å^3^	0.30 × 0.20 × 0.20 mm
*Z* = 4	

### Data collection

**Table:**

Enraf-Nonius CAD-4 diffractometer	*R*_int_ = 0.060
Radiation source: fine-focus sealed tube	θ_max_ = 25.3º
Monochromator: graphite	θ_min_ = 2.3º
*T* = 294(2) K	*h* = 0→10
ω/2θ scans	*k* = 0→11
Absorption correction: ψ scan(North *et al.*, 1968)	*l* = −12→12
*T*_min_ = 0.327, *T*_max_ = 0.382	3 standard reflections
1726 measured reflections	every 120 min
1620 independent reflections	intensity decay: 1%
769 reflections with *I* > 2σ(*I*)	

### Refinement

**Table:**

Refinement on *F*^2^	Secondary atom site location: difference Fourier map
Least-squares matrix: full	Hydrogen site location: inferred from neighbouring sites
*R*[*F*^2^ > 2σ(*F*^2^)] = 0.078	H-atom parameters constrained
*wR*(*F*^2^) = 0.166	*w* = 1/[σ^2^(*F*_o_^2^) + (0.050*P*)^2^ + 3.3*P*] where *P* = (*F*_o_^2^ + 2*F*_c_^2^)/3
*S* = 1.00	(Δ/σ)_max_ < 0.001
1620 reflections	Δρ_max_ = 0.48 e Å^−^^3^
100 parameters	Δρ_min_ = −0.51 e Å^−^^3^
Primary atom site location: structure-invariant direct methods	Extinction correction: none

### Special details

**Table:**

Geometry. All e.s.d.'s (except the e.s.d. in the dihedral angle between two l.s. planes) are estimated using the full covariance matrix. The cell e.s.d.'s are taken into account individually in the estimation of e.s.d.'s in distances, angles and torsion angles; correlations between e.s.d.'s in cell parameters are only used when they are defined by crystal symmetry. An approximate (isotropic) treatment of cell e.s.d.'s is used for estimating e.s.d.'s involving l.s. planes.
Refinement. Refinement of *F*^2^ against ALL reflections. The weighted *R*-factor *wR* and goodness of fit *S* are based on *F*^2^, conventional *R*-factors *R* are based on *F*, with *F* set to zero for negative *F*^2^. The threshold expression of *F*^2^ > σ(*F*^2^) is used only for calculating *R*-factors(gt) *etc*. and is not relevant to the choice of reflections for refinement. *R*-factors based on *F*^2^ are statistically about twice as large as those based on *F*, and *R*- factors based on ALL data will be even larger.

### Fractional atomic coordinates and isotropic or equivalent isotropic displacement parameters (Å^2^)

**Table:**

	*x*	*y*	*z*	*U*_iso_*/*U*_eq_	
Br	0.57689 (14)	−0.36443 (12)	0.40395 (11)	0.0876 (5)	
Cl	−0.1214 (3)	−0.4586 (3)	−0.3126 (3)	0.0960 (10)	
O	0.3720 (7)	−0.3636 (6)	0.1096 (6)	0.0663 (18)	
C1	0.5206 (10)	−0.2276 (10)	0.2666 (9)	0.064 (2)	
H1A	0.5247	−0.1379	0.3073	0.077*	
H1B	0.5933	−0.2292	0.2056	0.077*	
C2	0.3720 (10)	−0.2470 (10)	0.1918 (9)	0.064 (2)	
H2A	0.2995	−0.2589	0.2522	0.077*	
H2B	0.3443	−0.1667	0.1383	0.077*	
C3	0.2602 (12)	−0.3856 (11)	0.0161 (11)	0.071 (3)	
C4	0.1241 (11)	−0.3031 (10)	0.0054 (9)	0.066 (2)	
H4A	0.1144	−0.2325	0.0644	0.079*	
C5	0.0111 (12)	−0.3336 (11)	−0.0948 (10)	0.074 (3)	
H5A	−0.0765	−0.2832	−0.1003	0.089*	
C6	0.0200 (10)	−0.4318 (10)	−0.1849 (8)	0.061 (2)	
C7	0.1567 (11)	−0.5088 (10)	−0.1676 (10)	0.070 (3)	
H7A	0.1688	−0.5780	−0.2276	0.084*	
C8	0.2624 (10)	−0.4863 (10)	−0.0733 (9)	0.062 (2)	
H8A	0.3455	−0.5430	−0.0661	0.075*	

### Atomic displacement parameters (Å^2^)

**Table:**

	*U*^11^	*U*^22^	*U*^33^	*U*^12^	*U*^13^	*U*^23^
Br	0.1226 (10)	0.0711 (7)	0.0759 (7)	0.0158 (7)	0.0374 (6)	−0.0003 (7)
Cl	0.088 (2)	0.101 (2)	0.099 (2)	−0.0010 (18)	0.0109 (17)	−0.0052 (19)
O	0.092 (5)	0.052 (4)	0.067 (4)	−0.006 (4)	0.052 (4)	−0.013 (4)
C1	0.069 (5)	0.058 (5)	0.070 (5)	−0.001 (5)	0.022 (4)	−0.005 (5)
C2	0.072 (5)	0.051 (5)	0.074 (5)	−0.001 (4)	0.023 (4)	−0.011 (5)
C3	0.068 (5)	0.074 (6)	0.077 (5)	0.002 (5)	0.032 (5)	−0.004 (5)
C4	0.085 (6)	0.050 (5)	0.069 (5)	0.003 (4)	0.031 (4)	0.001 (4)
C5	0.075 (5)	0.074 (6)	0.079 (5)	0.010 (5)	0.032 (4)	0.010 (5)
C6	0.066 (5)	0.062 (5)	0.055 (4)	−0.001 (4)	0.008 (4)	0.019 (4)
C7	0.078 (6)	0.059 (5)	0.075 (5)	0.001 (4)	0.021 (4)	0.000 (5)
C8	0.062 (5)	0.055 (5)	0.073 (5)	0.008 (4)	0.021 (4)	0.005 (4)

### Geometric parameters (Å, °)

**Table:**

Br—C1	1.957 (9)	C3—C4	1.466 (13)
Cl—C6	1.719 (10)	C4—C5	1.378 (13)
O—C3	1.311 (11)	C4—H4A	0.9300
O—C2	1.418 (10)	C5—C6	1.342 (13)
C1—C2	1.466 (12)	C5—H5A	0.9300
C1—H1A	0.9700	C6—C7	1.440 (13)
C1—H1B	0.9700	C7—C8	1.281 (12)
C2—H2A	0.9700	C7—H7A	0.9300
C2—H2B	0.9700	C8—H8A	0.9300
C3—C8	1.347 (13)		
			
C3—O—C2	120.1 (8)	C5—C4—C3	117.9 (10)
C2—C1—Br	114.6 (6)	C5—C4—H4A	121.1
C2—C1—H1A	108.6	C3—C4—H4A	121.1
Br—C1—H1A	108.6	C6—C5—C4	123.8 (10)
C2—C1—H1B	108.6	C6—C5—H5A	118.1
Br—C1—H1B	108.6	C4—C5—H5A	118.1
H1A—C1—H1B	107.6	C5—C6—C7	115.1 (9)
O—C2—C1	109.8 (8)	C5—C6—Cl	121.4 (8)
O—C2—H2A	109.7	C7—C6—Cl	123.5 (8)
C1—C2—H2A	109.7	C8—C7—C6	122.9 (10)
O—C2—H2B	109.7	C8—C7—H7A	118.6
C1—C2—H2B	109.7	C6—C7—H7A	118.6
H2A—C2—H2B	108.2	C7—C8—C3	123.9 (10)
O—C3—C8	122.2 (9)	C7—C8—H8A	118.0
O—C3—C4	121.4 (10)	C3—C8—H8A	118.0
C8—C3—C4	116.3 (10)		
			
C3—O—C2—C1	−167.0 (8)	C4—C5—C6—C7	2.1 (14)
Br—C1—C2—O	−70.5 (8)	C4—C5—C6—Cl	−176.5 (7)
C2—O—C3—C8	172.7 (8)	C5—C6—C7—C8	0.2 (14)
C2—O—C3—C4	−8.7 (13)	Cl—C6—C7—C8	178.8 (8)
O—C3—C4—C5	−179.4 (9)	C6—C7—C8—C3	−2.9 (15)
C8—C3—C4—C5	−0.7 (13)	O—C3—C8—C7	−178.4 (9)
C3—C4—C5—C6	−1.8 (14)	C4—C3—C8—C7	3.0 (14)

### Hydrogen-bond geometry (Å, °)

**Table:**

*D*—H···*A*	*D*—H	H···*A*	*D*···*A*	*D*—H···*A*
C2—H2A···Cg1^i^	0.97	2.88	3.665 (3)	138

Symmetry codes: (i) *x*, −*y*+1/2, *z*−1/2.

## References

[bb1] Allen, F. H., Kennard, O., Watson, D. G., Brammer, L., Orpen, A. G. & Taylor, R. (1987). *J. Chem. Soc. Perkin Trans. 2*, pp. S1–19.

[bb2] Baggaley, K. H. & Watts, E. A. (1982). European Patent Application EP0049060.

[bb3] Enraf–Nonius (1989). *CAD-4 Software* Enraf–Nonius, Delft, The Netherlands.

[bb4] Harms, K. & Wocadlo, S. (1995). *XCAD4* University of Marburg, Germany.

[bb5] Menini, L. & Gusevskaya, E. V. (2006). *Appl. Catal. A Gen.* **309**, 122–128.

[bb6] North, A. C. T., Phillips, D. C. & Mathews, F. S. (1968). *Acta Cryst.* A**24**, 351–359.

[bb7] Sheldrick, G. M. (2008). *Acta Cryst.* A**64**, 112–122. 10.1107/S0108767307043930 18156677

[bb8] Spek, A. L. (2003). *J. Appl. Cryst.* **36**, 7–13.

[bb9] Zirngibl, L., Fischer, J., Jahn, U. & Thiele, K. (1988). *Ann. N. Y. Acad. Sci.* **54**, 63–73. 10.1111/j.1749-6632.1988.tb40389.x 3214095

